# Plasmonic Photothermal Nanoparticles for Biomedical Applications

**DOI:** 10.1002/advs.201900471

**Published:** 2019-07-22

**Authors:** Minho Kim, Jung‐Hoon Lee, Jwa‐Min Nam

**Affiliations:** ^1^ Department of Chemistry Seoul National University Seoul 08826 South Korea; ^2^ Department of Chemistry City University of Hong Kong Hong Kong SAR, P. R. China

**Keywords:** metal nanoparticles, phothermal effect, photothermal therapy, plasmonic nanoparticles, theranostics

## Abstract

Recent advances of plasmonic nanoparticles include fascinating developments in the fields of energy, catalyst chemistry, optics, biotechnology, and medicine. The plasmonic photothermal properties of metallic nanoparticles are of enormous interest in biomedical fields because of their strong and tunable optical response and the capability to manipulate the photothermal effect by an external light source. To date, most biomedical applications using photothermal nanoparticles have focused on photothermal therapy; however, to fully realize the potential of these particles for clinical and other applications, the fundamental properties of photothermal nanoparticles need to be better understood and controlled, and the photothermal effect‐based diagnosis, treatment, and theranostics should be thoroughly explored. This Progress Report summarizes recent advances in the understanding and applications of plasmonic photothermal nanoparticles, particularly for sensing, imaging, therapy, and drug delivery, and discusses the future directions of these fields.

## 1 Introduction

**Figure 1:**
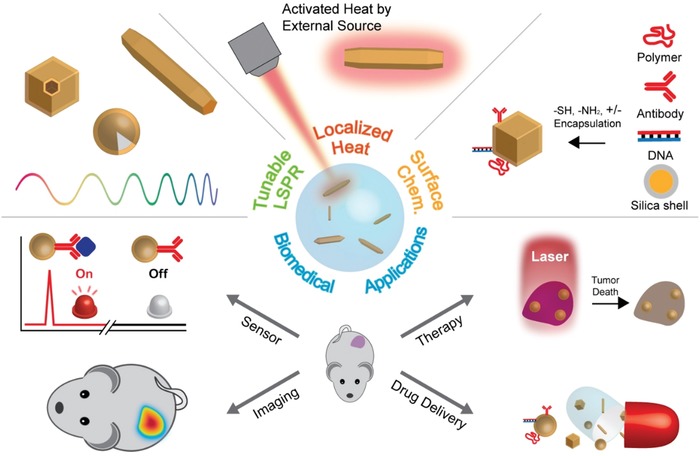
Representative features of photothermal nanoparticles and their biomedical applications.

Due to their larger optical cross‐sections, compared with those of organic dyes typically used for bioimaging and sensing, plasmonic nanoparticles have been extensively utilized for their light scattering as nanoantenna or contrast agents for surface‐enhanced Raman scattering,^1^ metal‐enhanced fluorescence,^2^ and optical imaging such as dark‐field^3^ and computed tomography (CT).^4^ Apart from radiative scattering of light, absorption of light by nanoparticles can also be nonradiatively relaxed and results in significant heat energy^5^ or photoluminescence.^6^ In particular, the conversion of light to thermal energy, known as the photothermal effect, by plasmonic nanomaterials has been extensively used for photothermal therapy applications.^7^ Since the discrete size and unique shape of nanoparticles are directly correlated with their plasmonic properties, particularly with localized surface plasmon resonance (LSPR), and the converted heat energy is highly localized near the nanoparticles, the plasmonic photothermal effect can be used as an efficient heat source for controllable and uniform thermal release at a specific excitation wavelength (**Figure** **1**). Thus, application of plasmonic photothermal nanomaterials has been extended to various biomedical applications, including biosensing,^8^ bioimaging,^9^ drug delivery,^10^ therapy^11^ as well as non‐biomedical applications, such as energy,^12^ chemical separation,^13^ nanofluidics,^14^ nanocatalysis,^15^ steam generation,^16^ and even synthesis,^17^ which all require and utilize specific and localized heat energy.

This progress report focuses on the fundamentals of plasmonic photothermal properties and the recent advances in plasmonic photothermal metallic nanoparticles, especially for biomedical applications. We first briefly introduce the optical properties and photothermal effect of plasmonic nanoparticles. We then highlight several key studies on the use of the photothermal effect in biosensing, bioimaging, therapy, and drug delivery applications. We conclude by outlining the ongoing challenges in this field and discuss future directions.

## 2 Plasmonic Properties and Materials

### 2.1 Optical Properties of Metallic Nanomaterials

The physical, chemical, and optical properties of metals are highly dependent on the spatial motion of the constituent electrons. In particular, when the dimensions of the metallic materials are much smaller than the wavelength of incident light (i.e., 1–100 nm), the spatial restrictions of electronic motion give rise to new properties that are quite different from those of the bulk materials (confinement effect).^18^ When a nanometer‐sized metallic particle is illuminated by light, the free electrons located on the particle surface are excited, and the local electron cloud is asymmetrically distributed over the metallic nanoparticle. The displacement of the electron cloud relative to the nuclei (i.e., charge separation) produces a coulombic restoring force between the negative electrons and the positive nuclei, which leads to a series of back‐and‐forth oscillations of the electron cloud on the particle surface.^18^, ^19^ This collective coherent oscillation of the conduction band electrons within metallic nanoparticles occurring at the metal/dielectric interface is termed localized surface plasmon (LSP).^20^ When the frequency of the incident light matches (resonance) with the LSP oscillation frequency of the plasmonic metal nanoparticles (e.g., gold, silver, and copper), the plasmonic nanoparticles strongly absorb the light, which generates highly amplified and localized electric fields in the vicinity of the particle surface. This resonant condition of LSP at a particular frequency of light is termed the LSP resonance (LSPR). In the LSPR condition, excited LSPs follow two different decay processes. Some of the absorbed light decays radiatively by emitting photons with the same frequency as the incident light (scattering), while some decays nonradiatively by converting into phonons (absorption).^21^ LSPs originate from the electronic motions localized in the plasmonic metal nanoparticles. Thus, LSPR is highly dependent on the factors affecting the density of electrons on the particle surface, such as size, shape, composition, dielectric properties of metal, and the surrounding medium.^18^, ^22^ These factors affect the absorption and scattering properties of plasmonic nanoparticles.

### 2.2 Extinction (Absorption and Scattering) and Plasmonic Heating

When plasmon oscillations are excited in a metal nanoparticle, the LSPR oscillations induce strong absorption and/or scattering of light, which reflects the observed color of the colloidal metal nanoparticle solution. According to the Gans theory, the cross‐sections of absorption (*C*_abs_), scattering (*C*_sca_), and total extinction (*C*_ext_) can be quantitatively derived as follows^18^, ^23^

(1) $$C_{\mathrm{abs}}=\frac{2\pi }{3\lambda }\varepsilon_{m}{}^{3/2}V\sum_{i}\frac{\varepsilon_{2}/{\left(n^{\left(i\right)}\right)}^{2}}{{\left(\varepsilon_{1}+\left[\left(1\;-\;n^{\left(i\right)}\right)/n^{\left(i\right)}\right]\varepsilon_{m}\right)}^{2}+\varepsilon_{2}{}^{2}}$$

(2) $$C_{\mathrm{sca}}=\frac{8\pi^{3}}{9\lambda^{4}}\varepsilon_{m}{}^{2}V^{2}\sum_{i}\frac{{\left(\varepsilon_{1}-\varepsilon_{m}\right)}^{2}+\varepsilon_{2}{}^{2}/{\left(n^{\left(i\right)}\right)}^{2}}{{\left(\varepsilon_{1}+\left[\left(1-n^{\left(i\right)}\right)/n^{\left(i\right)}\right]\varepsilon_{m}\right)}^{2}+\varepsilon_{2}{}^{2}}$$

(3) $$C_{\mathrm{ext}}\;=\;C_{\mathrm{abs}}\;+\;C_{\mathrm{sca}}$$

where λ is the wavelength of light, ε_m_ the dielectric constant of surrounding medium, ε the dielectric constant of the metal defined by ε = ε_1_ + *iε*_2_ (ε_1_ and ε_2_ indicate the real and imaginary parts of the dielectric constant, respectively), *V* the unit volume of the nanoparticle, and *n* ^(^ *^i^* ^)^ is the depolarization factor, expressed as follows

(4) $$n^{\left(a\right)}\;=\;\frac{1}{R^{2}\;-\;1}\left(\frac{R}{2\sqrt{R^{2}\;-\;1}}\ln\frac{R\;+\;\sqrt{R^{2}\;-\;1}}{R\;-\;\sqrt{R^{2}\;-\;1}}\;-\;1\right)$$

(5) $$n^{\left(b\right)}\;=\;n^{\left(c\right)}\;=\;\left(1\;-\;n^{\left(a\right)}\right)/2$$

where, *a*, *b*, and *c* (*a* > *b* = *c*) indicate a geometric factor (i.e., the three axes) of the nanoparticle (for instance, *n* ^(^ *^i^* ^)^ is equal to 1/3 for sphere‐shaped nanoparticle), and *R* the aspect ratio (i.e., *a*/*b*). Importantly, the LSPR occurs at ε_1_ = −(1 − *n* ^(^ *^i^* ^)^) × ε_m_/*n* ^(^ *^i^* ^)^, and the absorption, scattering, and total extinction can be strongly enhanced at such resonance conditions. The above equations reveal that the real part of the dielectric constant (ε_1_) determines the LSPR condition (i.e., resonance wavelength), whereas the imaginary part of the dielectric constant (ε_2_) contributes weakly to the LSPR condition and determines its bandwidth.^24^ More importantly, the cross‐sections of absorption and scattering largely depend on the dimensions of the nanoparticles; however, the factors affecting them are different—the *C*_sca_ is proportional to *V* ^2^, while the *C*_abs_ is linearly proportional to *V*. These factors are highly related to the changes in optical properties according to the nanoparticle size, which will be discussed below.

**Figure 2:**
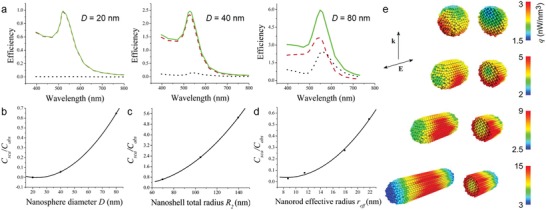
Size and shape‐dependent absorption, scattering, and extinction properties of plasmonic nanoparticles. a) Calculated spectra of optical efficiency for gold nanospheres with different diameters (*D*): 20 nm (left), 40 nm (middle), and 80 nm (right). The red‐dashed, black‐dotted, and green solid lines indicate the efficiencies of absorption, scattering, and extinction for gold nanospheres, respectively. As the size of nanosphere increases, the contribution of absorption and scattering to the total extinction decreases and increases, respectively. b–d) Size‐dependent changes in the relative ratio of scattering to absorption cross‐sections (*C*_sca_/*C*_abs_) of nanoparticles: b) gold nanosphere, c) gold nanorod, and d) silica–gold nanoshell. In case of the nanoshell, the total particle radius (*R*_2_) are varied (70, 105, and 140 nm) for a fixed ratio of the core radius (*R*_1_) to the total particle radius (*R*_1_/*R*_2_ = 0.857). In case of the nanorod, the effective nanorod radius (*r*_eff_) that determines the volume (*V*) of the nanorod [*r*_eff_ = (3*V*/4π)^1/3^] varied (8.74, 11.43, 17.9, and 21.86 nm) at a fixed aspect ratio of 3.9. Reproduced with permission.^26^ Copyright 2006, American Chemical Society. e) 3D mapping of the heat power density of different shaped colloidal gold nanoparticles (nanosphere and nanorod) at their respective LSPR conditions. Cross‐sectional images (right side) reveal the heating power density of the inner parts of each nanostructure. Reproduced with permission.^28^ Copyright 2009, AIP Publishing.

The optical features (e.g., absorption, scattering, and extinction) of the plasmonic metal nanoparticles depend on the wavelength of the light, and also on the size and shape of the nanoparticles.^22^ In case of the small‐sized gold nanoparticles (diameter <10 nm), the LSPR band is largely dampened because of the phase changes resulting from the higher rate of electron–surface collisions.^25^ As the size of the nanoparticles increases, the peak intensity of the LSPR band increases, and the LSPR band is broadened due to the dominant contributions from higher‐order electron oscillations.^25^ Such size‐dependent changes in absorption and scattering behavior was investigated by Jain, based on the Mie theory calculations.^26^ For the gold nanospheres of size 20 nm, the absorption was the dominant contributor to the total extinction, whereas the contribution of Mie scattering to the total extinction increased as the nanosphere size increased (**Figure** **2**a). Thus, the relative ratio of scattering to absorption (*C*_sca_/*C*_abs_) increased with increasing nanosphere diameter (Figure 2b), which is related to the increased radiative damping in larger nanoparticles.^26^ Apart from the gold nanospheres, different shapes of nanostructures, such as gold nanorod (i.e., geometry of a cylinder with two hemispheres) and silica–gold nanoshell (i.e., geometry of the closed shell formed on the silica core), also exhibit size‐dependent changes in absorption and scattering cross‐sections. The relative contributions of scattering increased as total volume of nanorod and total size of nanoshell increased (Figure 2c,d). These trends provide a diversity in the selection of suitable nanoparticles for the desired optical applications. In this scenario, smaller nanoparticles (<40 nm gold nanosphere and nanorod) are preferred as excellent photo‐absorbing agents for the photothermal effect‐based biomedical applications, while larger nanoparticles (>40 nm gold nanosphere and nanorod) are more proper in a biological imaging requiring a higher scattering efficiency.^26^, ^27^ In particular, in the case of silica–gold nanoshell, the relative sizes of the silica core and gold shell as well as the overall size of silica–gold nanoshell have a significant effect on the *C*_sca_ and *C*_abs_— with the fixed overall size of the silica–gold nanoshell, as the thickness of the gold shell becomes thinner (i.e., the larger the silica core), the absorption efficiency gets larger while the scattering efficiency gets lower.^26^

The optical properties of the plasmonic metal nanoparticles can also change markedly with shape as well as size. For an accurate comparison of the shape‐dependent changes in the optical properties, the size‐normalized cross‐sections (i.e., volumetric coefficients, defined by the optical cross‐sections per particle volume) were calculated for the different nanoparticle configurations, such as gold nanospheres, silica–gold nanoshells, and gold nanorods.^26^ Regardless of the particle dimensions (i.e., size), gold nanorods exhibited the volumetric absorption and scattering coefficient values an order of magnitude higher than those of nanospheres and silica–gold nanoshells, indicating that gold nanorods offer the most superior near‐infrared (NIR) absorption and scattering. Gold nanorods with a smaller volume and a higher aspect ratio are the best photo‐absorbers (i.e., higher volumetric absorption value), while those with a larger volume and higher aspect ratio are the best scattering contrast agents (i.e., higher volumetric scattering value). These shape‐dependent changes in the light‐absorption directly reflect the plasmonic heating efficiency of metallic nanostructures. Baffou et al. numerically investigated heat generation in plasmonic nanostructures (in the plasmonic resonance condition) using Green's dyadic method, and evaluated the geometric effect on the plasmonic heating process.^28^ To focus on the morphology effect and exclude the size effect, the same volume of gold nanospheres and gold nanorods were used as models. In case of a gold nanosphere excited at its LSPR, most of the area of the nanoparticle remained inactive and only the outer part of the nanoparticle facing the incoming light showed the plasmon‐induced heating generation (Figure 2e). However, in case of an elongated gold nanorod (with the same volume as the nanosphere), the entire volume of the nanostructure was more efficiently involved in the heating process and no longer suffered from the shielding effect that was evident for nanospheres (Figure 2e). This effect mainly reflects structure; the inner and outer parts of the particle are close to each other, so an incoming electric field can penetrate deeply inside the thin nanostructure and involve the entire volume of the nanostructure in plasmonic heating. These results show that small, flat, or elongated plasmonic nanostructures are more suitable for the light‐absorption and the subsequent heat generation, even with the same volume.

As mentioned above, the optical properties of the plasmonic metal nanoparticles can be controlled by varying size and shape, and these structural factors also affect the photothermal conversion efficiency. Wang et al. systematically investigated the photothermal conversion efficiency and the molar heating rate of plasmonic nanoparticles with gold nanoparticles with varying sizes and morphologies (nanorods and nanostars in this case).^29^ For both gold nanorods and nanostars, smaller nanoparticles showed higher photothermal conversion efficiencies while larger nanoparticles exhibited faster molar heating rate due to the larger molar extinction coefficients. Considering the effect of particle volume on photothermal conversion efficiency, gold nanostars exhibited a similar photothermal conversion efficiency compared with gold nanorods; however, gold nanostars showed a much higher molar heating rate than gold nanorods because of their large extinction and absorption efficiencies, indicating gold nanostars with a large volume is more promising for the photothermal heating than gold nanorods with the same longitudinal plasmon peaks.

### 2.3 Plasmonic Photothermal Effect

**Figure 3:**
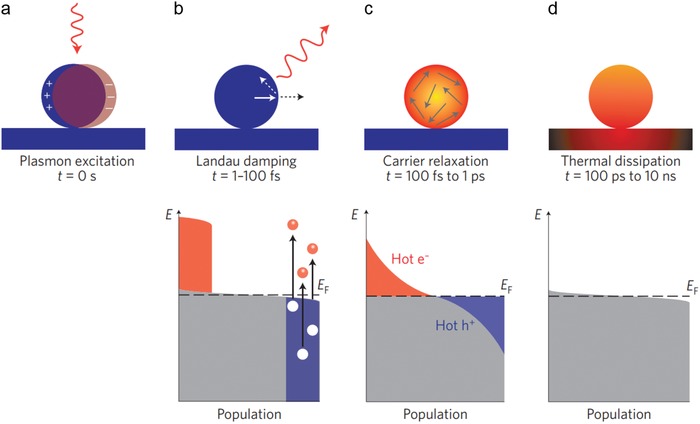
Photoexcitation and relaxation process of LSP on metallic nanoparticles. a) Photoexcitation of localized surface plasmon. b–d) Schematic illustrations of the population of the electronic states during relaxation of the photoexcited localized surface plasmon. The electronic states, hot electrons, and hot holes are depicted by grey, red, and blue, respectively. *E*_F_: Fermi energy. b) Landau damping. On a timescale of 1–100 fs, hot electron–hole pairs are created, and their energy is nonthermally distributed. c) Carrier relaxation. On a timescale of 100 fs to 1 ps, the energy of hot carriers (i.e., hot electrons and hot holes) are redistributed by the electron–electron scattering process. d) Thermal dissipation. On a timescale of 100 ps to 10 ns, the photoexcited energy transferred to the metallic lattice through electron–phonon collisions is released to the surroundings of the metallic nanoparticles in the form of heat. Reproduced with permission.^30^ Copyright 2015, Springer Nature.

Upon irradiating noble metal nanoparticles (e.g., gold, silver, copper) with external light at the appropriate wavelengths, free electrons on the nanoparticle surface are excited and conduction‐band electrons collectively oscillate at the same frequency (i.e., LSPR).[qv: 22a] The highly excited and energetic LSP can decay radiatively by reemission of light or nonradiatively by generating hot electrons.[qv: 21a,30] By transferring the energy of LSP to the electrons in the conduction band of the noble metal, these photoexcited highly energetic electrons have several effects, including photoemission, photochemistry, photodesorption, photocurrent, electrical doping, and local heating,^30^ which have been widely used in many fields such as photodetection,^31^ photovoltaics,^32^ and photocatalysts.^33^ As shown in **Figure** **3**, the photoexcited LSPs are relaxed in the following order: Landau damping, carrier relaxation, and thermal dissipation.^30^ When LSPs are photoexcited by external light at appropriate wavelengths (Figure 3a), hot electron–hole pairs are created, and the photoexcited electrons with a maximum energy corresponding to the excitation energy occupy energy states above the Fermi energy (*E*_F_) (Figure 3b). At these states, electron energy is nonthermally distributed with regard to Fermi–Dirac statics. The nonthermally distributed hot carriers (i.e., hot electrons and hot holes) are relaxed (redistributed) by the electron–electron scattering process without loss of the absorbed photon energy, leading to an internal electron thermalization (Figure 3c).^5^, ^34^ During the timescale of 100 ps to 1 ns, which corresponds to the final relaxation step after LSP excitation, the photoexcited energy above the *E*_F_ transfers to the metallic lattice through electron–phonon collisions.^30^ This relaxation step eventually induces thermal dissipation and releases the thermal energy to the surrounding medium (i.e., photothermal effect) (Figure 3d).[qv: 21a,30] During this external thermalization, the temperature of the electron gas and the lattice are steadily decreased, and the electron distribution in the conduction band of the metallic nanoparticle returns to its ground state prior to photoexcitation.^5^

The photophysical processes from light absorption (heating of plasmonic nanoparticles) to thermal dissipation (cooling of plasmonic nanoparticles) have been extensively studied using ultrafast laser spectroscopy.[qv: 5,24b,35] Since the volume of the nanoparticle lattice can change periodically when the nanoparticle is heated by laser pulses, a coherent vibrational mode can be excited during rapid heating of the nanoparticle lattice, which leads to transient oscillations of the absorption signal. From the coherent vibrational motion of gold nanoparticles (e.g., nanosphere and nanorod), Hartland et al.^36^ observed that vibrational periods were ≈5 and ≈50 ps for the gold nanospheres and nanorods, respectively. The vibrational period of the nanospheres displayed an inversely proportional correlation to the radius of the particles, while that of nanorods changed linearly with the length of the rods. This light‐absorption‐induced rapid heating of the plasmonic nanoparticle lattice should be carefully considered in applying the photothermal effect to biomedical fields. If the lattice heating due to the absorption of light is much faster than the cooling by the surrounding medium, the photothermal heat that accumulates within the lattice can result in the structural changes of nanoparticles, such as ablation,^37^ melting,^38^ or reshaping.^39^ Therefore, the lattice cooling should be efficient (i.e., slower rates of energy accumulation relative to lattice cooling) so that the temperature inside the nanoparticles raised by the light‐absorption is effectively transferred to the medium surrounding the nanoparticle, which can be used for photothermal effect‐based biomedical applications.

### 2.4 Plasmonic Photothermal Metallic Nanoparticles

In most biomedical applications that use plasmonic nanostructures, visible or NIR light is mainly used;^40^ thus, it is important to select appropriate nanostructures that exhibit higher absorption of visible or NIR light. In particular, NIR light (650–900 nm) has been widely used in biomedical applications because it can penetrate deep into the body due to the reduced absorption and scattering of photons by biological tissues (e.g., blood, water, melanin, and fat).^41^ Among the various materials, gold, silver, and copper have been mainly studied for photothermal effect‐based biomedical applications because their LSPR can cover most of the visible and NIR range,^42^ unlike aluminum, platinum, or palladium, which exhibit weak and broad LSPR bands in the ultraviolet region.^43^ Recently, the utilization of platinum and palladium nanoparticles to the photothermal therapy under irradiation of NIR light has been reported.^44^ It should be noted that the molar extinction coefficients of platinum and palladium nanoparticles in the NIR spectral range are typically 1–2 orders of magnitude lower compared to other promising nanoparticles (the molar extinction coefficients for the similar‐sized porous palladium nanoparticles,[qv: 44a] gold nanorods,^29^ and gold nanostars^29^ are ≈6.3 × 10^7^, ≈3.6 × 10^9^, and ≈5.8 × 10^8^ m ^−1^ cm^−1^, respectively), resulting in relatively low photothermal efficiency in the NIR region. Interestingly, the hexagonal palladium nanosheets exhibit a molar extinction coefficient of ≈4.1 × 10^9^ m ^−1^ cm^−1^, comparable to gold nanorods^45^ (≈5.5 × 10^9^ m ^−1^ cm^−1^), with a distinct LSPR band in the NIR region.^46^ This unusual optical features of palladium nanosheets stems mainly from an ultrathin 1.8 nm thickness with <10 atomic layers, and these nanoparticles showed very good in vitro photothermal cell‐killing efficacy under NIR irradiation, which reveals promising utilization of palladium nanostructures in the field of photothermal therapy.

In particular, gold is highly considered as the most suitable noble metal for biomedical applications because of its chemical/biological stability, low cytotoxicity in a biological environment, and diversely available surface functionalizations with various biological ligands such as DNA, proteins, and antibodies.^47^ Silver, which is likely to exhibit stronger and sharper plasmon resonance features than gold, can cause photothermal light‐to‐heat conversion more efficiently due to its superior optical features including larger extinction, absorption and scattering cross‐sections, and silver nanoparticles have been used as photothermal light‐to‐heat conversion transducers and antibacterial agents.^48^ However, since silver and copper may cause severe toxicity for in vivo applications and are not as chemically stable as gold,^49^ their use in clinical applications still requires more studies and advances to ameliorate the biocompatibility and stability in physiological environments. For this reason, this progress report focuses mainly on the biomedical utilization of gold nanomaterials for photothermal applications.

**Figure 4:**
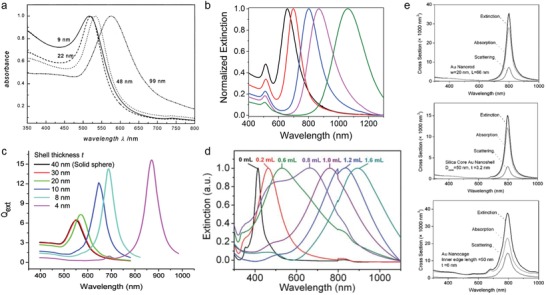
Size‐ and shape‐dependent tunability of optical properties of plasmonic nanoparticles. a) Ultraviolet–visible (UV–vis) absorption spectra of spherical‐shaped gold nanoparticles in water. All spectra were normalized at their LSPR absorption maxima (λ_max_; unit of nanometer). As the particle diameter increases, the plasmon absorption red‐shifts: λ_max_ = 517, 521, 533, and 575 nm for the 9, 22, 48, and 99 nm particles, respectively. Reproduced with permission.^50^ Copyright 1999, American Chemical Society. b) Surface plasmon absorption spectra of gold nanorods with different aspect ratios (i.e., length‐to‐width ratio). As the aspect ratio of gold nanorod increases (aspect ratios of 2.4, 2.7, 3.6, 4.4, and 6.1 for black, red, blue, magenta, and green spectra, respectively), the longitudinal plasmonic absorption band red‐shifts from the visible to the NIR region, while the transverse plasmonic absorption band rarely changes, regardless of the aspect ratio. Reproduced with permission.[qv: 51a] Copyright 2016, Royal Society of Chemistry. c) Tuning the LSPR wavelength of the 80 nm diameter core–gold nanoshells in water with different shell thickness (*t*): *t* = 40, 30, 20, 10, 8, and 4 nm. All the extinction efficiency (*Q*_ext_) spectra were calculated by using an extended Mie theory simulation. As the shell thickness decreases from 40 to 4 nm, the LSPR wavelength red‐shifts progressively from the visible to the NIR region and the extinction efficiency increases. Reproduced with permission.[qv: 51b] Copyright 2007, American Chemical Society. d) UV–vis extinction spectra of gold nanocages with the walls of different thicknesses and porosities. As the volume of HAuCl_4_ solution labeled on each curve increases (i.e., increasing the degree of galvanic replacement reaction), the LSPR peaks continuously red‐shifts from the visible to the NIR region due to the structural changes from the silver nanocube to the gold nanocage. e) Comparison of the absorption, scattering, and extinction spectra for gold nanorod (top), nanoshell (middle), and nanocage (bottom). All the optical spectra were obtained from the DDA calculation method, and the LSPR peaks were adjusted to exactly 800 nm for all the nanostructures. Reproduced with permission.[qv: 21b] Copyright 2006, Royal Society of Chemistry.

Considering that the optical resonance (i.e., LSPR) of the plasmonic nanoparticles should lie in the NIR spectral window for efficient biomedical applications, spherical gold nanoparticles are not suitable due to the limited tunability of their LSPR frequency: the relationship between nanosphere size and LSPR frequencies is limited in the visible region (520–580 nm) (**Figure** **4**a).^50^ When plasmonic nanoparticles change in shape from spherical to other morphologies (e.g., rod, shell, and cage), the LSPR can be tuned to be located in the NIR region.[qv: 21b,51] Rod‐shaped nanoparticles consisting of short and long axes exhibit two optical resonances: plasmonic oscillations along the short axis (transverse mode) and the long axis (longitudinal mode).^52^ The LSPR of nanorods strongly depends on the structural aspect ratio (i.e., length‐to‐width ratio), and the longitudinal plasmon mode (i.e., long‐axis LSPR wavelength) is red‐shifted from the visible to the NIR spectral region as the aspect ratio of nanorod increases, whereas the transverse mode is relatively insensitive to the aspect ratio (Figure 4b).[qv: 51a] In addition, the aspect ratio of gold nanorods can be precisely controlled by the amounts of silver ions or gold seeds added during the growth step.^53^ These varied changes in optical behavior according to the shape of nanostructures can be understood by the Gans theory,^54^ which explains the optical properties of ellipsoidal particles. This provides promising opportunities for nanorods in biomedical applications utilizing the NIR optical window. According to the previous literatures,^55^ it has been shown that the LSPR absorption maximum (λ_max_; unit of nanometer) is linearly dependent on the aspect ratio (*R*) as follows

(6) $$\lambda_{\max}=\left(52.95R-41.68\right)\varepsilon_{0}+466.38$$

where ε_0_ is the dielectric constant of the surrounding medium.

The synthetic mechanism for the formation of rod‐shaped nanoparticles has been proposed by several groups.^56^ For example, the head group of hexadecyltrimethylammonium bromide used as a surfactant selectively binds to specific crystal facets of the gold seeds. This selective surface‐blocking leads to different growth kinetics according to the crystal facets (i.e., anisotropic growth), resulting in formation of rod‐shaped nanoparticles.[qv: 56b] Further details are provided elsewhere.^57^ Various methods of synthesizing gold nanorods have been described, such as the template‐directed method,^58^ electrochemical method,^59^ seed‐mediated growth method,[qv: 53a] and photochemical reduction method.^60^

Another plasmonic nanostructure responding NIR spectral window is the metal nanoshell.[qv: 51b,61] Halas et al. were the first to synthesize the nanoshell structure through the surface coating of a thin gold shell on silica beads.[qv: 61a] Typically, silica beads are first prepared using the Stöber method, followed by surface modification to produce a monolayer of amino‐terminated silane. The small gold colloids (1–2 nm) are then suspended with the silica beads. The gold colloids attach to the amine group on the silica beads. The additional gold is deposited on the surface of the silica beads, resulting in the formation of a gold nanoshell structure having a silica core. The LSPR peaks of gold nanoshells are red‐shifted from the visible to the NIR region as the shell thickness decreases,[qv: 51b,61a] because the coupling between the inner and outer shell surface plasmons increases (Figure 4c).[qv: 51b,61b] These optical features are strongly dependent on the shell thickness‐to‐core radius ratio (i.e., the LSPR frequency decreases near‐exponentially as the shell thickness‐to‐core radius decreases), and are independent of the core material, shell metal, nanoshell size, or surrounding medium, which provides a universal and convenient way to design nanoshells for the required optical resonance conditions. Compared with the gold nanorods (Figure 4b), the gold nanoshells have higher sensitivity in the structure‐dependent tunability of the plasmonic properties (Figure 4c). For example, a small variation of shell thickness (from 8 to 4 nm) can largely shift the LSPR peak from ≈680 to ≈870 nm.

Similar to the gold nanorods and nanoshells, gold nanocages also show a good optical response over the NIR spectral region, and their LSPR conditions are tunable by controlling the wall thickness and porosity.[qv: 21b,62] Generally, the gold nanocages are prepared through the well‐known wet‐chemical galvanic replacement reaction, and silver nanocubes are used as a sacrificial template. Due to the different standard potential between the gold and silver, the silver atoms on the surface of silver nanocube continuously react with the gold ions and replace them, resulting in formation of nanocage structures^63^

$$3\mathrm{Ag}\left(s\right)+{\mathrm{AuCl}}_{4}{}^{-}\left(\mathrm{aq}\right)\to \mathrm{Au}\left(s\right)+3{\mathrm{Ag}}^{+}\left(\mathrm{aq}\right)+4{\mathrm{Cl}}^{-}\left(\mathrm{aq}\right)$$

In a typical replacement reaction, at the early stage, small holes are generated on the surface of silver nanocubes by the reaction between the silver nanocubes and the gold ions. The continuous replacement reactions result in the epitaxial deposition of the replaced gold atoms on the silver nanocube surface to form a thin shell. Simultaneously, nanobox‐shaped gold–silver alloy structures are formed via the diffusion of silver atoms into the gold shell. Finally, as more gold ions are introduced, the corners of the gold–silver alloy nanoboxes are dealloyed and etched, leading to the formation of porous gold nanocages (i.e., nanoboxes with truncated corners). By adjusting the molar ratio between silver nanocubes and gold ions, the thickness of the walls and size of the holes in the nanocages can be variously controlled. This galvanic replacement reaction method can be extended to synthesize the various shapes of porous/hollow structures according to the shape of sacrificial templates (e.g., nanospheres, nanorods, and nanotubes).^63^ Importantly, the LSPR peaks of gold nanocages can be easily tuned according to the different thicknesses and porosities of the walls (Figure 4d).[qv: 21b,62] As the structure changes from silver nanocubes to gold nanocages by the continuous galvanic replacement reaction, the positions of the LSPR peaks shift widely from the visible to the NIR region (Figure 4d), indicating the usability for the biomedical applications. Furthermore, due to the 3D opened porous/hollow structure, the gold nanocages can be used as new class of efficient drug carriers, which can be potentially utilized for drug delivery or controlled drug release.^64^

To utilize the gold nanostructures for photothermal effect‐based biomedical applications, it is necessary to properly design the nanostructures so that the light‐to‐heat conversion is efficient. In particular, it is critical that the nanostructures be designed to have a high absorption cross‐section and a high ratio of absorption over total extinction, because the photothermal effect of metallic nanoparticles is fundamentally originated from the light‐absorption property. Based on this requirement, the NIR‐response metallic nanoparticles, including nanorods, nanoshells, and nanocages, are the most promising candidates for the photothermal effect‐based biomedical applications. Figure 4e shows the absorption, scattering, and extinction spectra of gold nanorods, nanoshells, and nanocages calculated using the discrete dipole approximation (DDA) method.[qv: 21b] For the calculations, the structural models of the nanoparticles were as follows: gold nanorods with a width of 20 nm and a length of 66 nm; gold nanoshells with a shell thickness of 3.2 nm on the silica cores with a diameter of 50 nm; and gold nanocages with a wall thickness of 6 nm and an inner edge length of 50 nm. In addition, the LSPR peaks of these nanostructures were adjusted to 800 nm. In all structures, the absorption cross‐section over total extinction was larger than the scattering cross‐section, and gold nanorods and nanocages showed larger absorption and scattering cross‐sections than nanoshells, with more than two times stronger extinction cross‐sections. These results indicate that gold nanorods and nanocages with higher absorption cross‐sections are more efficient light‐to‐heat plasmonic converters than nanoshells. Specifically, Maestro et al. investigated the absorption efficiency of different‐shaped gold nanostructures exhibiting intense LSPR at around 808 nm.^65^ The optical properties including extinction, absorption, and scattering cross‐sections as well as absorption efficiency and single particle optical cross‐sections per unit mass are summarized in **Table** **1**. In this study, the gold nanorods showed the smaller extinction (*C*_ext_), absorption (*C*_abs_), and scattering cross‐sections (*C*_sca_) than silica–gold nanoshells and gold nanocages, however, these results are mainly caused by a relatively smaller dimension of gold nanorods compared to that of other structures. To exclude the effect on the optical properties due to the size of the nanostructures, the *C*_ext_, *C*_abs_, and *C*_sca_ of nanostructures were normalized by the mass. In this case, the gold nanorods exhibited the highest values of single particle optical cross‐sections per unit mass among other nanostructures (Table 1), which means that even with a relatively small amount of gold, the gold nanorods can exhibit similar levels of optical properties. Because the photothermal effect is basically related with light‐absorption process, the light‐to‐heat conversion efficiency of plasmonic nanostructures is highly dependent on the absorption efficiency (Φ_abs_; defined by fraction of absorption and extinction cross‐sections, *C*_abs_/*C*_ext_) of nanostructures. Together with higher values of gold nanorods regarding all optical cross‐sections per unit mass, the absorption efficiency of gold nanorods was also higher than that of other nanostructures: Φ_abs_ of 0.95 ± 0.04, 0.63 ± 0.02, and 0.68 ± 0.03 for nanorods, nanocages, and nanoshells, respectively. Importantly, the absorption cross‐sections of these nanostructures (*C*_abs_ = 10^3^–10^4^ nm^2^) are 5–6 orders of magnitude higher compared to those of conventional organic dyes (e.g., indocyanine green (ICG) of *C*_abs_ = 1.66 × 10^−2^ nm^2^ at ≈800 nm).^66^ Thus, they are sufficient to convert light to heat with much lower laser energies, and are minimally invasive in the photothermal effect‐based biomedical applications.

**Table 1:** Optical characteristics of the plasmonic gold nanoparticles with different geometries

Geometry	Dimensions [nm]	[Au] [g]^a)^	*C*_ext_ [nm^2^]	*C*_abs_ [nm^2^]	*C*_sca_ [nm^2^]	Φ_abs_
			*C*_ext_/[Au] [nm^2^ g^−1^]^b)^	*C*_abs_/[Au] [nm^2^ g^−1^]^b)^	*C*_sca_/[Au] [nm^2^ g^−1^]^b)^	
Gold nanorods	8 ± 1 width	2.8 × 10^−17^	4.5 × 10^3^	4.3 × 10^3^	2.0 × 10^2^	0.95 ± 0.04
	29 ± 5 length		1.6 × 10^20^	1.5 × 10^20^	7.1 × 10^18^	
Silica–gold nanoshells	120 ± 5 Si core	1.0 × 10^−14^	1.5 × 10^4^	1.0 × 10^4^	5.0 × 10^3^	0.68 ± 0.03
	10 ± 1 Au shell		1.5 × 10^18^	1.0 × 10^18^	5.0 × 10^17^	
Gold nanocages	47 ± 3 edge	4.7 × 10^−16^	5.0 × 10^4^	3.1 × 10^4^	1.9 × 10^4^	0.63 ± 0.02
	4 ± 1 wall thickness		1.1 × 10^20^	6.6 × 10^19^	4.0 × 10^19^	

^a)^ The [Au] indicates the gold mass per nanoparticle that corresponds to different geometries

^b)^ The single particle optical cross‐section per unit mass was calculated using the values of *C*_ext_, *C*_abs_, *C*_sca_, and [Au] given in the literature.^65^

In addition to the excellent optical properties mentioned above, the gold nanorods have practical advantages over other structures in biomedical applications. Unlike gold nanocages and silica–gold nanoshells, the gold nanorods can be uniformly synthesized in a reproducible manner by wet‐chemistry without complex synthetic steps at room temperature.[qv: 53a,67] In contrast, the synthesis of gold nanocages usually requires relatively high temperature (≈100 °C) during the galvanic replacement reaction to prevent the formation of AgCl precipitates that affect the epitaxial deposition of gold atoms on the surface of the silver template and to promote the high interdiffusion rate between silver and gold which affects the structural homogeneity and reproducibility of the final product.^63^ In the case of silica–gold nanoshells, the requirement of complex multistep synthesis (synthesis of silica particles and gold nanoparticles, chemical modification of silica surface, attachment of gold nanoparticles on silica surface, and additional deposition of gold to form silica–gold nanoshells) is tedious and time‐consuming.^68^ Particularly for in vivo biomedical applications, it is important to design nanostructures to exhibit strong optical properties with higher absorption efficiency and small size if possible. Smaller nanoparticles can be more easily excreted from bodies, which can minimize undesired toxicity induced by nanoparticles left in bodies.^69^ In these aspects, the small gold nanorods with superior optical properties are considered as suitable in vivo photothermal agents. It should be noted that the surfaces of all the plasmonic nanostructures mentioned above are composed of gold, which can be versatilely modified with various kinds of ligands (e.g., thiols, phosphines, and amines).^47^ For this reason, it is possible to change the surface charge of nanoparticles and to introduce various functional groups, which eventually can provide long‐term stability in physiological environments and prevent particle aggregation by protecting the nanoparticle surface (e.g., PEGylation). Further, such a versatility in surface modification is highly applicable in modifying ligands (e.g., DNA or antibodies) that selectively recognize bioanalytical targets or cells of interest.

Recently, photothermal nanostructures with an optical response in the second NIR window (1000–1400 nm wavelength, NIR‐II window) have emerged for biomedical applications to replace the traditional first NIR window (700–1000 nm wavelength, NIR‐I window). Since light absorption and scattering are less for biological samples, particularly for in vivo environment, with NIR‐II window than with NIR‐I window,^41^, ^70^ the NIR‐II window requires very low external light intensity, which is far below the skin tolerance threshold set by the American National Standards Institute (ANSI), and allows for deep skin penetration possible, making the NIR‐II window more suitable for in vivo applications.^71^ Furthermore, the NIR‐II window is relatively safe for practical purposes compared to the NIR‐I window because it has a higher value of maximum permissible exposure (MPE) to laser; the MPEs of skin to laser are 0.33 and 1 W cm^−2^ for the NIR‐I and NIR‐II windows, respectively.^72^ For these reasons, the NIR‐II‐window‐responsive nanostructures, such as quantum dots^73^ and carbon nanotubes,^74^ and small‐molecule fluorescent dyes[qv: 70b,75] have been developed; it should be noted that they have been mainly developed as imaging agents without plasmonic photothermal effect. Various types of NIR‐II‐window‐responsive plasmonic nanoparticles (e.g., gold/silver alloyed nanocages,^76^ gold nanorings,^77^ gold nanorod‐in‐shell nanostructures,^78^ gold nanoechinus,^79^ and gold nanodumbbells^80^) have been reported, and these structures hold potentials to be used for biomedically useful photothermal applications.

## 3 Bio‐Applications of Plasmonic Photothermal Nanoparticles

### 3.1 Sensing

Early detection, rapid and reliable assay, and high‐mobility are essential in the discovery and identification of new outbreak contagions, such as the recent Ebola and Zika crises, and crucial for preventing the spread of such diseases and urgently treating infected patients. We discuss several recent research studies using the plasmonic photothermal effect directly or indirectly in biosensing applications.

**Figure 5:**
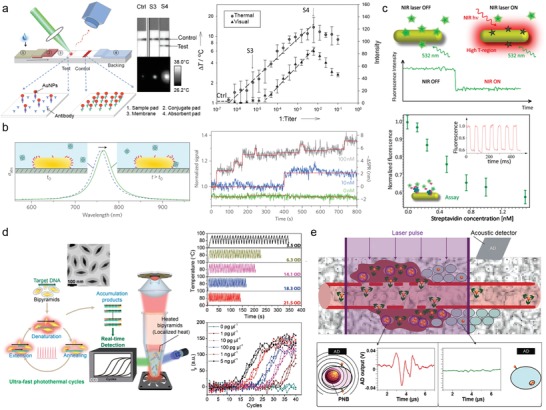
Plasmonic photothermal nanoparticle‐based biodiagnostics. a) Scheme and results of thermal contrast‐based lateral flow tests for immunoassays. While the visual contrast of AuNPs is insufficient, thermal contrast can detect the target at low concentrations with clear signals. Reproduced with permission.^82^ Copyright 2012, Wiley‐VCH. b) Photothermal detection of single molecules using AuNRs without labelling or amplification in real time. The steps in the photothermal time trace data show real‐time single‐molecule binding and unbinding events. Reproduced with permission.^8^ Copyright 2012, Springer Nature. c) Photothermal fluorescence quenching‐based immunoassay method. The data plots show normalized fluorescence emission as a function of streptavidin concentration. Each data point corresponds to the average of five replications by multiple on/off cycles of the NIR laser (inset). Reproduced with permission.^89^ Copyright 2016, American Chemical Society. d) Plasmonic photothermal nucleic acid amplification using AuBPs. The data shows temperature profiles of 30 thermocycles with different concentrations of particles and real‐time amplification curves obtained from different concentrations of target template. Reproduced with permission.^92^ Copyright 2017, American Chemical Society. e) Scheme of PNB diagnostics of residual microtumors and cancer cells in vivo. The acoustic signal from PNBs (red line) indicates a single cancer cell in solid tissue [normal cells (green line)]. Reproduced with permission.^94^ Copyright 2016, Springer Nature.

Lateral flow tests based on capillary materials are the most simple, inexpensive, and widely used medical diagnostic methods to detect target analytes for home and point‐of‐care testing.^81^ Latex or gold nanoparticles (AuNPs) are typically used as colored particles for naked‐eye detection. However, the sensitivity of this method is typically limited to ≈µm. Qin et al. significantly improved the sensitivity of AuNP‐based lateral flow tests by introducing thermal contrast as a detection method (**Figure** **5**a).^82^ They used a general sandwich‐type immunoassay on nitrocellulose membrane to detect the target antigens of interest, which consisted of AuNPs and two antibodies modified on the membrane. In the presence of target analytes, the AuNPs accumulated at the test line. Compared to naked‐eye colorimetric detection, the thermal contrast from the photothermal effect of AuNPs generated 32‐fold greater sensitivity in signal in cryptococcal antigen (CrAg) testing. They further showed that the sensitivity could be increased up to 10 000 folds by introducing nanostructures with better light‐to‐heat conversion efficiency, such as gold nanorods (AuNRs). More recently, Zhao et al. reported that photoacoustic signal‐based detection improved the limit of detection for CrAg by 100‐fold compared to colorimetric lateral flow tests.^83^ The photoacoustic signal is the sound wave generated from the light‐to‐heat conversion of plasmonic nanomaterials and subsequent periodic thermal expansion of the surrounding media. The authors also showed that the photoacoustic detection method can be applied to enzyme‐linked immunosorbent assays (ELISAs) as a signaling probe and improve the detection limit by 142‐fold compared to conventional methods.^84^

Since single‐molecule sensitivity can determine the heterogeneity of various biomolecular interactions, single molecule detection has been an intense research area in medicine, biosensors, and molecular biology.^85^ Scattering based‐dark‐field microscopy is the most common technology in this field used to detect LSPR changes of single nanoparticles by either binding or unbinding of biomolecules,^3^ but the sensitivity of single molecule events in this method is still limited.^86^ Recently, Li et al. showed that human papillomavirus (HPV) can be detected at 6.5 × 10^−18^ m with dark‐field microscopy by counting the number of AuNRs encoded with target‐capturing DNA strands. They used a sandwich‐type assay between a magnetic bead and an AuNR to capture target DNA by a two‐step DNA hybridization process.^87^ Zijlstra et al. reported a label‐free and real‐time single‐molecule plasmonic detection method using AuNRs and photothermal microscopy (Figure 5b).^8^ AuNRs functionalized with biotins (red‐colored) at the tip were used to monitor the binding events of proteins with different molecular weights and concentrations. Upon binding of proteins, the longitudinal plasmon mode of AuNRs is redshifted due to the locally increased index of refraction. Consequentially, it changes the absorption cross‐section of the AuNRs at the fixed wavelength of the heating beam, and therefore the temperature of the AuNRs is changed. This temperature change can be detected with photothermal microscopy, which can detect AuNPs with diameters as small as 1.4 nm.^86^ Therefore, this technique has improved sensitivity with a higher signal‐to‐noise ratio compared with dark‐field spectroscopy. This is because the LSPR of smaller nanoparticles can be highly influenced by the absorbed molecules. Using this assay method, the authors showed single‐binding and unbinding protein interactions in real time by measuring the photothermal signals, and improved the sensitivity of the single molecule assay by more than two orders of magnitude compared to the conventional scattering‐based method.

Fluorescence labeling and detection has been a powerful method in various biomedical applications due to many analytical advantages.^88^ Pellegrotti et al. reported a novel method of fluorescence quenching with plasmonic photothermal effect used for bioassay (Figure 5c).^89^ They demonstrated that the fluorescence emission of organic dyes can be significantly reduced near nanoparticles by light‐induced thermal energy, which is different from the well‐known fluorescence suppression by metallic nanoparticles in close proximity due to direct energy transfer of the dyes to particles. In the presence of streptavidin, Alexa Fluor 546‐labeled biotins can approach the surface of biotin‐labeled AuNRs, and the fluorescence signals can be quenched by the thermal energy generated near the particle surface. They quantified the reduced fluorescence emission in the reaction solution to determine the concentration of analytes, and achieved a limit of detection as low as ≈250 × 10^−12^ m.

Nucleic acid amplification by polymerase chain reaction (PCR) is a highly sensitive and reliable method of characterizing genetic information, and remains the gold standard in sensitive nucleic acid detection.^90^ However, there remains a critical need for new technologies to address current limitations of this technology, such as long procedural time, heavy and expensive instrumentation, enzyme‐based amplification process, and high energy consumption.^91^ Lee et al. demonstrated a plasmonic photothermal PCR assay based on the light‐assisted photothermal heating of plasmonic nanoparticles (Figure 5d).^92^ In their method, Au bipyramid nanoparticles (AuBPs) were evenly dispersed in solution and acted as heating nanoreactors to absorb the photonic energy and convert it to heat energy. They showed that 30 thermocycles from 72 to 95 °C can be finished in <2.5 min using a light‐emitting diode (LED) as a light source. Using a viral M13mp18 amplicon and a laboratory‐built LED device, they demonstrated that the targeted sequences could be quantitatively amplified and detected in real time with a substantial dynamic range of 5 ng µL^−1^ to 1 pg µL^−1^, comparable to conventional real‐time PCR. Moreover, they successfully extended the photothermal system to other related nucleic acid amplification methods, such as rolling circle amplification and multistep digestion, which require a constant temperature for many hours.

Microscopic residual disease (MRD) is a significant problem in many cancers that requires complete tumor resection to prevent lethal recurrence.^93^ Lukianova‐Hleb et al. reported an intraoperative surgical method to detect and eliminate residual microtumors using nanobubbles induced from plasmonic nanoparticles (Figure 5e).^94^ Plasmonic nanobubbles (PNBs) are nanosized vapors with nanosecond lifespans generated near nanoparticles from the absorption of pulsed light, and cause an acoustic signal from the periodic thermal expansion of the surrounding media. The authors used head and neck squamous cell carcinoma as a model system because of its high recurrence. AuNPs were conjugated for efficient targeting of cancer cells with the panitumumab monoclonal antibody. Upon accumulation of the nanoparticles in the tumor region, the acoustic signals from PNBs could be detected from the targeted cancer cells containing nanoparticle aggregates. They successively detected 3 to 30 residual cancer cells located 4 mm deep in the mouse model in real time. Furthermore, they achieved 100% tumor‐free survival in resectable MRD and a twofold improved survival rate in unresectable MRD in real time by using PNB‐guided surgery.

For practical use of plasmonic sensing systems as diagnostic platforms, the miniaturization of sensing devices that enable rapid, portable, wearable, accurate, and active real‐time monitoring is critical and beneficial. Recent advances in microfluidic lab‐on‐a‐chip technology have shown the potential to miniaturize existing biosensing systems.^95^ However, there is still a need for these miniaturized devices to reliably detect, read, analyze, transmit and display results.[qv: 95a,96] Based on the general analysis method, many efforts are currently underway to develop analytical biosensor systems based on smartphones such as microscope images, colorimetry, electrochemical and electrochemical luminescent biosensors. For example, Berg et al. reported a portable smartphone‐based enzyme‐linked immunosorbent assays system, consisting of a 3D‐printed optomechanical attachment to hold and illuminate a 96‐well plate using a light‐emitting‐diode (LED) array. Using the camera of the smartphones for signal reading, they showed both qualitative and quantitative measurements for detection signals with high accuracy.^97^ Barbosa et al. developed a portable colorimetric and fluorescence quantitative sandwich immunoassay detection system for prostate specific antigen by integrating a magnifying lens and light source into a smartphones to read the intensity of the fluorescence signal from a miniaturized microfluidic system.^98^ Wang et al. reported a self‐referenced portable nanoplasmonic imaging platform for colorimetric biomolecule sensing and demonstrated colorimetric sensing with a refractive index change due to its SPR and LSPR phenomena for the glucose and human cardiac troponin I (cTnI), showing better detection limit than the plate‐read or UV–vis spectrometer.^99^ In addition, there has been efforts to miniaturize and integrate the thermal imaging camera in the smartphone,^100^ which may be benefits in miniaturizing the thermal contrast‐base detection method. All these efforts to miniaturize sensors and integrate small sensors into smartphones and advances in various plasmonic nanomaterials will promote and stimulate the development of new types of photothermal sensing methods and devices with new sensing principles that can be practically useful and offer different ways of sensing with unconventional functionalities.

### 3.2 Imaging

**Figure 6:**
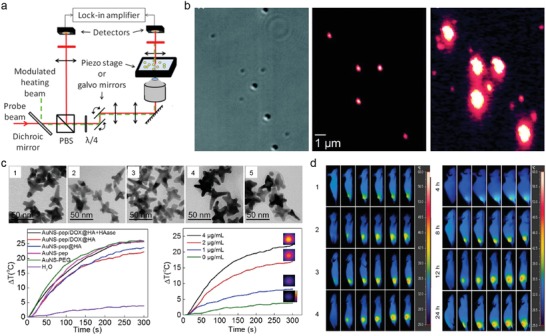
Photothermal microscope and thermal contrast images. a) Typical experimental setup for photothermal imaging. PBS: polarizing beam splitter; λ/4: quarter wave plate. Reproduced with permission.^101^ Copyright 2014, The Royal Microscopical Society, published by John Wiley and Sons. b) Differential interference contrast image (left) and two photothermal images (middle and right) of sample mixture with 300 nm latex spheres, and 80 and 10 nm gold spheres. In photothermal images, the intensity of the heating laser was 30 kW cm^−2^ (middle) and 1.5 MW cm^−2^ (right), respectively. Reproduced with permission.^86^ Copyright 2002, The American Association for the Advancement of Science. c) Transmission electron microscope images of gold nanostar (AuNS) with different surface functionalization: 1) unmodified AuNS, or AuNS modified with 2) PEG, 3) pep, 4) pep@HA, and 5) pep/DOX@HA. PEG: poly(ethylene glycol); HAase: hyaluronidase. (Bottom left) Temperature changes of nanoplatform solution with NIR irradiation (808 nm, 1 W cm^−2^). The concentration of Au was 4 µg mL^−1^. (Bottom right) Au concentration‐dependent temperature changes of AuNS‐pep/DOX@HA solution with NIR irradiation (808 nm, 1 W cm^−2^). Insets show photothermal images of the AuNS‐pep/DOX@HA solution at the final temperature. d) (Left) In vivo photothermal imaging of mice injected intravenously with different solution: 1) PBS, 2) AuNS‐PEG, 3) AuNS‐pep@HA, and 4) AuNS‐pep/DOX@HA (left). All images were acquired with NIR laser irradiation (808 nm, 1 W cm^−2^) at 12 h postinjection. (Right) In vivo photothermal imaging of mice intravenously injected with AuNS‐pep/DOX@HA irradiated with NIR laser (808 nm, 1 W cm^−2^, for 0, 1, 2, 3, 4, 5 min) at different time points (4, 8, 12, 24 h) after injection (right). Reproduced with permission.[qv: 104a] Copyright 2016, Elsevier.

Photothermal effect, which produces thermal contrast upon nonradiative plasmon decay, was recently used for imaging plasmonic nanoparticles, called photothermal imaging (PTI). In general, PTI requires two beams: a heating beam and a probe beam (**Figure** **6**a).^101^ Once the LSP of the nanoparticle is excited by the heating beam, the temperature of the nanoparticles and their surroundings increases, resulting in changes in their refractive index.^101^, ^102^ This photothermal effect‐induced variation of refractive index can be detected using an additional nonresonant laser source, called a probe beam.^101^, ^102^ In the PTI setup, these two beams are coaligned and focused in the same region of the sample using a microscope objective lens,[qv: 102a] and the detected variations by the probe beam are recorded with a photodiode connected to a lock‐in amplifier, providing a high signal‐to‐noise ratio.^101^, ^102^ Moreover PTI can further improve the signal‐to‐noise ratio with high sensitivity by choosing the probe beam wavelength at which the nanoparticles have minimum absorption.[qv: 102b]

It is known that Rayleigh scattering‐based dark‐field microscopy requires nanoparticles at least 40 nm in diameter for imaging, because the scattering signal of metallic nanoparticles decreases in proportion to the sixth power of the nanoparticle diameter, and its signal should be distinguishable from the strong background.^86^ By contrast, the absorption is much more dominant for small metallic nanoparticles over scattering, and the absorption decreases in proportion to only the third power of the nanoparticle diameter.^86^ PTI is thus more sensitive compared to the scattering‐based technique and its sensitivity is sufficiently high to detect nanoparticles with an absorption cross‐section of a few Å^2^.^101^ In 2002, Boyer et al. reported far‐field detection of nanometer‐sized metal particles using a photothermal method that combined high‐frequency modulation and polarization interference contrast (Figure 6b).^86^ In differential interference contrast (DIC) images, latex spheres with 300 nm diameters and AuNPs with 80 nm diameters were clearly seen due to the strong scattering signals, however, gold spheres with 10 nm diameters were not visible. In the case of photothermal images where a very low heating power (<100 µW), the 80 nm AuNPs were visible at the same location in the DIC image. However, the latex particles disappeared because of their nonlight‐absorbing property, and the 10 nm AuNPs remained invisible. When the heating power was increased (≈5 mW), the 10 nm AuNPs were clearly visible, whereas the 80 nm AuNPs provided very strong signals and saturated the detection capability. In addition, the photothermal image was free of background noise in biological samples because the absorption background from biomolecules is negligible. Berciaud et al. improved the sensitivity of the photothermal method by designing a photothermal heterodyne imaging system that combined a heating beam generating time‐modulated variations of the refractive index around an absorbing nanoobject and a probe beam producing a frequency‐shifted scattered field.^103^ They demonstrated exceptional detection of individual AuNPs down to a diameter of 1.4 nm (67 gold atoms) with a high signal‐to‐noise ratio, and showed 2 orders of magnitude improved sensitivity.

Despite their high sensitivity and long‐term stability, the conventional optical setup (i.e., requirement of two beams) in photothermal microscopy is limited for biological imaging because parallelization of the matrix detector, a charged coupled device (CCD), is not possible due to its lock‐in detection scheme.^101^ Due to this intrinsic limitation, photothermal microscopy requires a scanning technique that is not suitable for rapid wide‐range biological imaging.^101^ To overcome this limitation, a thermographic camera (also called an infrared camera or thermal imaging camera) was used recently to detect local temperature increases induced by the photothermal effect and directly observe the location of target nanoparticles in an in vivo model.^104^ Chen et al. synthesized a mitochondria‐targeting nanoplatform by coencapsulating peptide‐modified gold nanostars (AuNS‐pep) and doxorubicin (DOX) in a hyaluronic acid (HA) protective shell (Figure 6c).[qv: 104a] The different nanoplatforms containing an AuNS rapidly increased in temperature under NIR irradiation (808 nm) regardless of their surface functionalization. Importantly, the location of nanoplatform accumulation in the targeted tumor region was directly observed by in vivo PTI (Figure 6d). After 12 h postintravenous injection of nanoplatforms into the tumor‐bearing mice, the tumors were irradiated by an 808 nm laser at 1 W cm^−2^ for 5 min, and thermal images were recorded using an infrared camera. All mice treated with AuNS‐based nanoplatforms showed a local temperature increase at the tumor region, and time‐dependent accumulation of nanoplatforms at the tumor region was successfully monitored by PTI.

Besides Au nanostructures, graphene,^105^ carbon dot (CD),^106^ iron oxide nanoparticles,^107^ and upconversion nanoparticles,^108^ as well as their hybrid structures,^109^ have been used as PTI agents. Generally, as compared with plasmonic metal nanoparticles, nonmetal nanomaterials such as graphene, carbon dot, iron oxide, and upconversion nanoparticle are relatively difficult to precisely tune their optical properties in a straightforward manner to have the light‐absorption maxima in the NIR region. In addition, their extinction and absorption cross‐sections are considerably lower than plasmonic metal nanoparticles (typically, ≈3–4 orders of magnitude lower).^11^ Because the absorption efficiency (*C*_abs_/*C*_ext_) is a critical parameter for high photothermal efficiency, the lower absorption efficiency leads to inefficient PTI, which requires relatively strong external light intensity and longer exposure time for the effective PTI. In addition, plasmonic metal nanoparticles can enhance the total heat generation by clustering nanoparticles. Due to plasmonic coupling, one nanoparticle can feel an electric field induced by other nanoparticles that are plasmonically activated.^110^ Therefore, when plasmonic nanoparticles are clustered, the interactions between plasmonic nanoparticles can generate more heat than individually separated nanoparticles without plasmonic coupling. Given the fact that nanoparticles can be locally accumulated at the targeting region in PTI applications, localized plasmonic metal nanoparticles can induce PTI efficiency than nonplasmonic nanomaterials. Obviously, particle size and shape effect along with particle surface charge/modification effect should be also considered together, and all these affect biodistribution, targeting efficiency, circulation time, and excretion in a collective manner. Although the noble metal nanoparticles exhibit the improved properties in PTI based on their superior optical characteristics, the size of nanoparticles showing the optimized optical properties can be relatively larger than nonmetal PTI agents, which may limit their utilization in practical in vivo applications (e.g., inefficient excretion from body after use in vivo).^69^

Up to now, various types of plasmonic nanoparticles have been applied to PTI, and PTI has garnered much attention in biomolecular imaging due to its potential in sensitivity, real‐time monitoring, rapid recording with simple instruments and convenient use. However, in terms of practical use, the precise design and synthetic realization of a nanostructure that specifically maximizes plasmonic functionality of interest and suitability for the targeted biomedical application is needed. In order to apply the photothermal effect more efficiently in the field of imaging, utilization of photoacoustic effect‐based imaging modality (i.e., photoacoustic tomography, PAT) as well as direct thermal imaging via a thermographic camera is highly desired.^111^ When the energy of nonionizing laser pulse is absorbed by biological tissue or nanoparticles, those light‐absorbing materials convert the light energy into heat by transient thermoelastic expansion, resulting in produce wideband ultrasonic waves, called as photoacoustic effect.^112^ It should be noted that the photothermal and photoacoustic effects are highly correlated in that they both occur based on a light‐to‐heat conversion process, but PTI and PAT are distinguished in that the detecting signals are different (i.e., local temperature and ultrasonic wave). Through this “light in‐sound out” effect, the spatial resolution and imaging depth of the PAT can reach as much as several tens (or hundreds) of micrometers and several centimeters, respectively.^113^ However, when compared to other noninvasive imaging modalities that have an excellent imaging depth for in vivo applications,^114^ such as magnetic resonance imaging (MRI), CT, positron emission tomography (PET), and single photon emission computed tomography (SPECT), the imaging depths of PTI and PAT are still relatively shallow. In addition, PAT generally requires pulse energies of mJ level, which may degrade the light‐absorption features of plasmonic nanoparticles by causing structural damages (e.g., ablation, melting, and reshaping).[qv: 113b] In this respect, the development of nanostructures that maintain their structural rigidity and chemical and biological stability in harsh optical/physiological environments and the structural design of nanoparticles that can sufficiently produce heat even under lower external‐light‐energy are highly urgent for both PTI and PAT. Recently, to improve the efficiency of bioimaging and acquire more information concurrently, PTI has also been widely utilized in practical multimodal imaging (i.e., accurate visualization of anatomical structures and tumor location)^114^, ^115^ combined with other imaging tools, such as optical fluorescence,^116^ MRI,^117^ CT,^118^ PET,^119^ and SPECT.^120^ Furthermore, PTI can be combined with photothermal therapy (PTT) under the same light radiation; thus, it can offer not only easy accessibility for multimodal molecular imaging but also an efficient tool for real‐time imaging‐guided therapy.[qv: 112c,117b,121]

### 3.3 Therapy

PTT utilizes the accumulation of plasmonic nanoparticles at targeted tumors. External light absorbed by the nanoparticles causes a local temperature increase at the tumor, resulting in selective hyperthermia and irreversible damage of tumor tissue while avoiding damage to healthy tissue.^122^ PTT is a highly efficient and unique method for disease treatment compared with conventional therapeutic methods because it exhibits spatiotemporal selectivity, with the advantages of high sensitivity, less side effects, noninvasiveness, fast and effective treatment, and low cost.^123^ Fundamentally, PTT is a light‐mediated treatment, thus an appropriate choice of external laser stimulating plasmonic nanoparticles for use inside the body is important for enhancing in vivo therapeutic efficiency. For this reason, an NIR laser exhibiting low absorption or scattering by biological tissues such as blood, water, melanin, and fat is widely used for PTT, as it can penetrate deep into the targeted tissue to reach the embedded plasmonic nanoparticles.^124^

**Figure 7:**
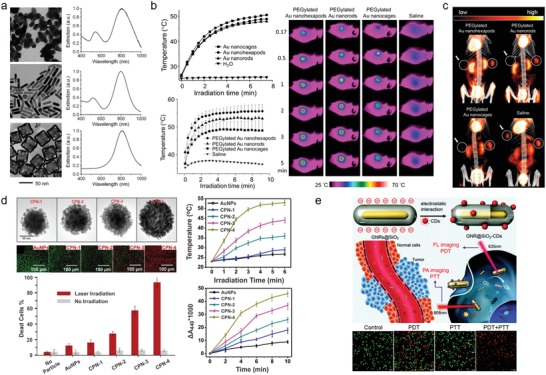
Various shapes of plasmonic nanoparticles for PPT/PDT applications. a) Transmission electron microscope images and UV–vis spectra of aqueous suspensions of plasmonic gold nanostructures with different morphologies: nanohexapods (top), nanorods (middle), nanocages (bottom). b) Plots of temperature rise as a function of laser irradiation time (top left) and plots of average temperature increase within the in vivo tumor region as a function of laser irradiation time (bottom left), and thermographs of tumor‐bearing mice after photothermal treatment for different periods of time (right). c) ^18^F‐flourodeoxyglucose PET/CT images of tumor‐bearing mice intravenously administrated with aqueous suspensions of PEGylated gold nanostructures or saline. Tumors on the left side (white circle + arrow) were treated with laser irradiation while those on the right side (white circle) were not treated. Reproduced with permission.^125^ Copyright 2013, American Chemical Society. d) Transmission electron microscope images of CPNs with different length and density of nanopetals, synthesized by increasing amounts of gold precursor from CPN‐1 to CPN‐4. Plots of temperature rise (top right) and the generation of ^1^O_2_ (bottom right) as a function of laser irradiation time for different gold nanoparticles, and cell death with or without laser irradiation (bottom left). Green and red fluorescence indicate live and dead cells, respectively. Reproduced with permission.[qv: 123b] Copyright 2014, American Chemical Society. e) Scheme of GNR@SiO_2_‐CDs for dual‐modal PTT/PDT therapy. GNR: gold nanorod. Fluorescence images of calcein acetoxymethyl ester and propidium iodide costaining of cancer cells incubated with GNR@SiO_2_‐CDs under laser irradiation: 635 nm (PDT), 808 nm (PTT), and combination (PDT + PTT). Green and red fluorescence indicate live and dead cells, respectively. Reproduced with permission.[qv: 109b] Copyright 2016, Royal Society of Chemistry.

Wang et al. reported a comparative study of the efficacy of photothermal cancer treatment according to the shape of nanoparticles (**Figure** **7**a).^125^ Synthesized gold nanostructures showed strong absorption of NIR light (≈800 nm) and rapid increase of solution temperature (Figure 7b). Irradiation with an NIR laser to tumor‐bearing mice injected with polyethylene glycol(PEG)‐protected gold nanostructures rapidly elevated the temperature near the tumor region to ≈50 °C, which was sufficient to kill cancer cells selectively. Among the gold nanostructures with different morphologies, nanohexapods (55.7 ± 2.4 °C) showed the highest photothermal conversion efficiency in vivo compared with nanorods (53.0 ± 0.5 °C) and nanocages (48.7 ± 3.5 °C). At 24 h after photothermal treatment, ^18^F‐flourodeoxyglucose uptake was remarkably decreased in tumors irradiated with an NIR laser compared with the contralateral nonirradiated tumors, indicating that glycolic activity of tumors was completely destroyed by PTT (Figure 7c). Quantitative analysis revealed that ≈90% of tumor metabolism was reduced by tumor‐selective PTT in vivo.

Interestingly, the photothermal effect itself indirectly enhances therapeutic efficacy, in terms of targeting efficiency. For example, photothermal local heating increases both vascular and cell membrane permeability, resulting in highly enhanced cellular uptake of photothermal nanoparticles in the targeted tissue.^126^ This increases the accumulation of nanoparticles in target cells while lowering the required dosage; thus, the therapeutic efficiency is enhanced at a lower dosage.^7^ Although PTT alone is sufficient for medical treatment, synergetic therapies with different therapeutic methods such as photodynamic therapy (PDT),^127^ radiotherapy,^128^ immunotherapy,^129^ and chemotherapy^130^ have been recently investigated to enhance the therapeutic efficacy of PTT. Among various therapeutic modalities, PDT is closely related to the optical properties of metallic nanoparticles. PDT is a site‐selective treatment that induces cell death through a process of converting normal tissue oxygen (^3^O_2_) into highly cytotoxic reactive oxygen species, such as singlet oxygen (^1^O_2_) and other free radicals.^131^ In general, ^1^O_2_ is generated by organic photosensitizers (PSs) such as rose bengal and phthalocyanine;^132^ however, those PSs are generally limited in clinical use because of their small absorption cross‐sections as well as photo‐induced degradation and enzymatic degradation.^133^ In contrast, metallic nanoparticles are promising PSs for PDT due to their excellent photostability, high absorption cross‐sections, and superior resistance to photo‐induced degradation and enzymatic degradation than conventional organic dyes.^134^ Typically, AuNRs exhibited much higher ^1^O_2_ generation capability under two‐photon excitation at 808 nm than traditional PSs such as rose bengal and ICG because of large two‐photon absorption action cross‐sections, ≈2 orders of magnitude larger than organic PSs.^134^ The generation of ^1^O_2_ caused by metallic nanostructures is mainly occurred by an electron transfer process from metals to ^3^O_2_ and followed by activation of ^3^O_2_ for eventual conversion to ^1^O_2_.^135^ Energetic electrons (called as hot‐electrons) involved in the activation of ^3^O_2_ are generated by the nonradiative relaxation process of photoexcited plasmons (Figure 3) and high‐energy electrons transfer into the 2π* antibonding O–O orbital to create O_2_^−·^and further produce ^1^O_2_ and ^·^OH,[qv: 135a] and the efficiency of PDT by nanoparticles is thus directly related to plasmonic properties of nanoparticles (i.e., efficient generation of hot electrons). Namely, both PDT characteristics and PTT characteristics are caused by the nonradiative relaxation process of the photoexcited plasmons, and these depend on the light‐absorption properties of nanoparticles. Therefore, given their operating mechanism, it is expected that the design of plasmonic nanoparticles to increase the efficiency of PDT will be similar with that of PTT as mentioned in Section 2.4 — more precisely, in case of PDT, not only hot electron generation efficiency but also hot electron transfer efficiency should be simultaneously considered.[qv: 21a]

Thanks to the excellent optical properties of the plasmon nanoparticles as described above, Kumar et al. synthesized plasmonic core–petal nanoparticles (CPNs) and successfully conducted dual phototherapeutic treatments (PTT and PDT), even without conventional organic PSs.[qv: 123b] Using an oxidative nanopeeling chemistry by gold chloride‐induced oxidative disassembly and rupture of the polydopamine layer on the Au core, the CPNs were formed in a high yield through the controlled anisotropic growth of petal structures on Au cores (Figure 7d). In comparison with spherical AuNPs, the visible/NIR light‐absorbing CPNs showed higher temperature increases of solution, when irradiated with a 785 nm laser. Due to the plasmonic electron transfer‐induced activation of ^3^O_2_, the CNPs highly produced ^1^O_2_ under laser excitation, and the generation of ^1^O_2_ was monitored by an *N*,*N*‐dimethyl‐4‐nitrosoaniline (RNO)‐histidine colorimetric assay. Because CPNs can efficiently induce photothermal effect and simultaneously induce PTT and PDT at the same wavelength, CPNs can kill cancer cells more efficiently using low laser power and short laser exposure time. Similarly, hybrid nanostructures with combinations of gold and other materials, such as CDs,[qv: 109b] graphene/photosensitizers,[qv: 121b] or upconversion nanoparticles,^136^ can also significantly improve the therapeutic efficiency of PTT (Figure 7e).

Considering the advantageous feature of NIR‐II window for biomedical applications (i.e., the higher value of MPE to laser and the reduced absorption and scattering of photons for in vivo environment enabling deeper penetration of external light in biological tissues), the NIR‐II‐window‐active plasmonic nanoparticles are promising for in vivo PTT applications. Vijayaraghavan et al. reported the multibranched gold nanoechinus (AuNEs) exhibiting photodynamic and photothermal therapeutic effects in both the NIR‐I and NIR‐II windows.^79^ Upon 915 nm (NIR‐I) and 1064 nm (NIR‐II) light irradiations, the B16F0 melanoma tumors in mice were completely destructed via PDT and PTT. Importantly, due to the extraordinarily high extinction coefficient of AuNEs (≈0.69 × 10^12^ m ^−1^ cm^−1^ at 915 nm and ≈0.74 × 10^12^ m ^−1^ cm^−1^ at 1064 nm) that are about 7–9 orders higher than conventional organic dyes, the PDT and PTT could be successfully conducted with much lower dosage of nanomaterials and irradiation time, even with ≈2–3‐fold lower laser power intensity than the standards set by ANSI for skin burning. Tsai et al. developed plasmonic gold nanorod‐in‐shell structures as a light‐absorption agent in the NIR‐I and NIR‐II windows for in vivo PTT.^78^ By introducing the galvanic replacement reaction on the gold/silver–core/shell nanorods, the gold nanorod‐in‐shell structures containing a nanogap between core and shell were obtained, which eventually exhibited strong absorption at the NIR‐I and NIR‐II spectral windows. Interestingly, the light‐absorption property of gold nanorod‐in‐shell structures was dependent on the gap size between core and shell: the gold nanorod‐in‐shell structures with a narrower gap (≈2 nm) exhibited a higher absorption band at the NIR‐II spectral window than those with the wider gap (≈4 and 6.5 nm), resulting in higher efficacy in the photothermal destruction of cancer cells (LLC/LL2 lung cancer cells) under irradiation of 1064 nm diode laser (NIR‐II). Apart from the NIR‐II‐window‐active metallic nanoparticles, a plasmonic hybrid nanostructure consisting of metal (gold) and semiconductor (copper chalcogenides) (Au–Cu_9_S_5_) was also reported for in vitro and in vivo PTT.^72^ Due to the coupling of the collective hole oscillation in the heavily doped semiconductors with the surface‐enhanced near‐field of metal nanoparticles, the Au–Cu_9_S_5_ hybrids exhibited 50% increase in the absorbance at 1064 nm (NIR‐II) compared to the pure Cu_9_S_5_ nanoparticles. The Au–Cu_9_S_5_ hybrids exhibiting large optical absorption cross section (≈1.3 × 10^8^ m ^−1^ cm^−1^) and high photothermal transduction efficiency (37%) showed excellent capability for both in vitro and in vivo PTT in the NIR‐II spectral window with a laser power density of 0.6 W cm^−2^, which is lower than the laser safety standards. However, most NIR‐II‐window‐responsive plasmonic nanoparticles have bimetallic compositions, which can be difficult to synthesize in a high yield, are relatively unstable and might cause high in vivo toxicity. In addition, many nanoparticles still require laser power above the skin tolerance threshold, and are too large (>300 nm) to be used for in vivo applications.

In order to make PTT nanoparticles clinically useful, the structural design of nanoparticles to improve photothermal heat conversion efficiency is key, but how efficiently nanoparticles can be “delivered” to the target cells (i.e., tumors) should be carefully considered because the delivery efficiency is directly related to the efficiency of selective tumor‐treatment by PTT. The delivery efficiency of nanoparticles to the target tumor site is affected by several factors including tumor‐specific‐targeting efficiency, circulation time and biodistribution of nanoparticles in body, and these factors are highly governed by the size, shape, surface coating and charge of nanoparticles.^137^ Recently, Wilhelm et al. conducted a multivariate analysis on the compiled data (the identified 224 relevant manuscripts were obtained from SciFinder and Google Scholar databases using “nanoparticle delivery” as a search term from 2005 to 2015) and revealed how the physicochemical parameters of nanoparticles (e.g., materials, size, shape, and surface charge), tumor models, and cancer types affect the delivery efficiency (%ID, defined as the percentage of nanoparticles that reached the target tumor compared to the injected dose (ID)) of nanoparticles.^138^ According to this, inorganic materials, such as gold, iron oxide, silica and quantum dots, showed higher %ID than organic materials, such as liposomes, polymers, hydrogels and dendrimers (0.8 and 0.6%ID for inorganic and organic materials, respectively). In particular, gold exhibited a higher %ID among other inorganic materials: 1.0, 0.6, 0.4, and 0.9%ID for gold, iron oxide, silica, and quantum dots, respectively. With respect to particle size (hydrodynamic diameter), the %ID was higher in the case of a relatively small size: 0.7, 0.7, 0.6, and 0.4%ID for <10, 10–100, 100–200, and >200 nm particles, respectively; and the rod‐shaped nanoparticles showed higher %ID than other nanoparticles: 0.8, 0.7, and 0.6%ID for rod, spherical, and plate or flake particles, respectively. Considering that gold nanorods with small volume and high aspect ratio can exhibit higher light‐to‐heat conversion efficiency due to high absorption efficiency and also considering the corresponding size and shape regime can result in relatively higher delivery efficiency in vivo, we can conclude that the smaller gold nanorods are more promising for in vivo PTT among other structures. In addition, when the surface charge (i.e., zeta potential) is relatively neutral or small (−10 to 10 mV), the %ID was higher than negative (<‐10 mV) and positive (>10 mV) cases: 0.7, 0.5, and 0.6%ID for neutral, negative, and positive cases, respectively; and active targeting strategy showed higher %ID than passive targeting strategy: 0.9 and 0.6%ID for active and passive targeting strategies, respectively.^138^ In conclusion, PTT efficiency can be further increased by attaching ligands that selectively recognize the target tumors to the surface of small‐sized gold nanorods and by adjusting their surface charge to be relatively neutral. To minimize unexpected potential toxicity induced by nanoparticles remaining in the body, the nanoparticles administered in vivo should be clearly excreted from body after use. The excretion efficiency of nanoparticles depends on the size, shape and surface coating of the nanoparticles,^69^ and the most critical factor on the excretion efficiency is particle size^139^ – smaller particles should work better for nanoparticle‐based in vivo PTT.

PTT is considered as a potential alternative to the treatment of diseases in the future because of its noninvasiveness, high sensitivity, and less side effect together with low cost and fast treatment. However, the fact that the delivery efficiency is still below 1%^138^ indicates that the clinical translation of nanoparticles for the use of PTT has been limited, particularly in practical use for human. The smaller delivery efficiency means that a larger dose is required in practical applications, and most of the administrated nanoparticles will remain outside the target cells (i.e., interaction with off‐target tissues), thus greatly reducing the efficiency of the treatment. Such an inefficiency is directly related to the critical problem increasing potential toxicity through off‐targeted nanoparticle residues in body, and from a commercial point of view, it may be a cost‐ineffective method in that it requires a higher dose. Since mass‐manufacturing of nanoparticles is required for widespread use of nanoparticles, it is necessary to realize the uniform and reproducible synthesis of the targeted nanoparticles in a large scale. In this respect, in order to greatly improve the treatment efficiency even with a small dose of nanoparticles by increasing the delivery efficiency, researches should focus on exploring the pharmacokinetics behavior of the administrated nanoparticles under the various physiological environments in body and probing precise the mechanism regarding the interactions between nanoparticles and human tissues. In addition, for meeting various users' demands, an optimized nanoparticle design that is capable of stably, uniformly, and reproducibly exhibiting an excellent light‐to‐heat conversion efficiency even with varying laser wavelengths, particularly in the wide NIR spectral range from NIR‐I to NIR‐II, is required.

### 3.4 Drug Delivery

**Figure 8:**
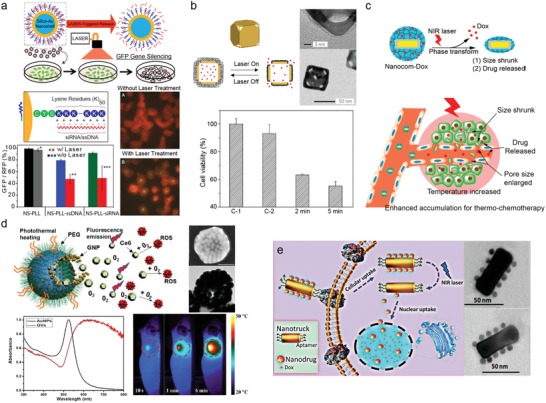
Photothermal nanostructures for drug delivery. a) siRNA and ssDNA delivery system using Au nanoshell (NS). Fluorescence images show H1299 cells incubated with NS‐PLL‐ssDNA. Upon laser irradiation, ssDNA tagged with Alexa Fluor 488 is released, resulting in brighter green fluorescence due to elimination of quenching. The histogram shows downregulation of green fluorescent protein (GFP) in H1299 GFP/red fluorescent protein (RFP) cell line by antisense ssDNA and siRNA. Percent GFP/RFP fluorescence at 18 h (6 h after laser treatment). Reproduced with permission.[qv: 140b] Copyright 2012, American Chemical Society. b) Schematic illustrating the controlled‐release system of AuNC. Cell viability test: (C‐1) cells pulsed laser irradiated for 2 min without AuNCs; (C‐2) cells laser irradiated for 2 min with DOX‐free AuNCs; and (2/5 min) cells laser irradiated for 2 and 5 min with DOX‐loaded AuNCs. Reproduced with permission.[qv: 64c] Copyright 2009, Springer Nature. c) Synthetic process and NIR laser‐induced targeted thermo‐chemotherapy of the nanocomposite. Reproduced with permission.^143^ Copyright 2014, American Chemical Society. d) Photosensitizer (Ce6)‐loaded gold vesicles (GVs) for trimodality fluorescence/thermal/photoacoustic imaging guided synergistic photothermal/photodynamic cancer therapy. Thermal images show tumor‐bearing (red circles) mice laser‐irradiated post injection of GV‐Ce6. Reproduced with permission.^147^ Copyright 2013, American Chemical Society. e) Cell‐targeted photocontrolled nuclear‐uptake nanodrug delivery system for cancer therapy. Reproduced with permission.^149^ Copyright 2014, American Chemical Society.

Targeted delivery of drugs or genes is integral to improving treatment efficiency and decreasing dosages to reduce unwanted side effects. Due to their tunable optical properties and highly localized heat, plasmonic metallic nanomaterials have been widely used in drug delivery as nanocarriers with high spatiotemporal resolution in order to remotely release drugs by external light modulation, and photothermal therapy can be combined with drug delivery. Huschka et al. reported the photothermal‐based release of oligonucleotides with Au nanoshells (**Figure** **8**a).^140^ They used a poly‐l‐lysine (PLL) peptide with positively charged lysine residues to electrostatically load single‐stranded DNA (ssDNA) or double‐stranded short‐interfering RNA (siRNA) with negative charges on their phosphate backbone. They demonstrated controlled delivery with continuous wave NIR laser irradiation and monitored the time‐dependent release process with fluorescently tagged oligonucleotides. Using the green fluorescent protein(GFP)‐labeled NCI‐H1299 human cancer cell line, they showed successful cellular uptake and gene silencing potential in vitro.[qv: 140b] Wang et al. reported a method to load and release drugs using AuNRs and DNAs, using the intercalating property of DOX with double‐stranded DNAs.^141^ DNAs loaded with the drug were modified on the AuNRs with electrostatic interaction. They demonstrated that a combination of photothermal heat and released drug by light resulted in higher cell death compared to free drug in an in vitro assay with 4T1 breast cancer cells. This method also significantly reduced the growth of primary tumors and suppressed lung metastasis in vivo in the orthotropic 4T1 mouse model.

Hybrid nanocomposite structures have been studied to combine the photothermal property of plasmonic nanomaterials and thermoresponsive property of polymers for controlled drug release.^10^ In order to efficiently design a composite for in vivo applications, the low critical solution temperature (LCST) of the polymers must be higher than the body temperature, but lower than the hyperthermia temperature required for both effective drug loading and release, and must provide circulation stability.^142^ Yavuz et al. developed porous hallow Au nanocages (AuNCs) covered with copolymers [poly(*N*‐isopropylacrylamide and acrylamide)] with an LCST of 39 °C (Figure 8b).[qv: 64c] They demonstrated the controlled in vitro release of alizarin‐PEG and DOX from the polymer‐coated hollow nanocages with NIR laser irradiation. Similar strategies using a thermosensitive polymer was adopted to silica‐coated AuNRs for targeted drug release by other groups (Figure 8c).^143^ Mesoporous silica‐encapsulated AuNRs were also studied for their cancer thermo‐chemotherapeutic capability.^144^ Due to the high drug loading capacity and structural stability of AuNRs from the high porosity and encapsulation of the silica shell, respectively, the composite structures could simultaneously deliver heat and anticancer drugs for improved cancer therapy. In other studies, liposome and Au shell hybrid structures have been reported to show improved therapeutic efficacy due to increased biocompatibility.^145^

In addition to nanoparticles and nanocomposites, self‐assembled plasmonic nanostructures have drawn interest for drug delivery and therapy applications because of the unique physical and chemical properties that result when they combine into a single specific nanostructure.^146^ For example, drugs can be loaded into the interior space of the assembled structures, and the optical properties can be tuned to the NIR region by the plasmon couplings of the assembled particles in close proximity, depending on the assembled size, geometry, and composition. With intelligent design of the assembly and disassembly process, these structures can potentially optimize the balance between long circulation time and rapid clearance from the body. Vesicles assembled from AuNPs or AuNRs coated with an amphiphilic block copolymer have been reported by several groups. Lin et al. fabricated multifunctional gold vesicles with PEG‐poly(styrene)‐coated spherical AuNPs that were loaded with the hydrophobic photosensitizer Ce6 to generate reactive oxygen species (ROS), which kill cancer cells (Figure 8d).^147^ The disassembled process by light‐induced heat resulted in efficient release of hydrophobic drugs at the specific tumor region. Due to strong absorption by the plasmon coupling from neighboring AuNPs, the gold vesicles showed potential for trimodal NIR fluorescence/thermal/photoacoustic image‐guided photothermal and photodynamic therapy in both in vivo and in vitro models of breast cancer. Similarly, Song et al. reported dissociable ultrasmall gold vesicles (≈60 nm) with AuNRs (8 nm × 2 nm) coated with PEG and poly(lactic‐*co*‐glycolic acid) for increasing the photothermal properties, tumor uptake, and clearance from the body.^148^ In another example, Qiu et al. used DNA self‐assembly to develop an NIR‐responsive nuclear‐uptake nanotruck for drug‐resistant cancer therapy (Figure 8e).^149^ In order to synthesize the core–satellite nanostructures for delivery, DNA‐modified AuNPs loaded with drugs (nanodrugs) were assembled on the side face of DNA‐modified silver–gold nanorods by DNA hybridization. The end faces of nanorods were modified with cell type–specific internalizing aptamers for targeting. Once the assembled nanostructures entered the cells, core–satellite nanostructures were disassembled inside the cell by photothermal heat and delivered the nanodrugs to the nucleus. Due to the small size of satellite nanodrugs, the nuclear uptake can be easily induced without nuclear localization signal peptides, thus, anticancer drugs can be efficiently transferred to the nuclei to kill cancer cells.

To date, a variety of different stimuli, including temperature, pH, magnetic fields, and light, have been applied to nanomaterial‐based drug delivery systems for triggering the release of drugs.[qv: 22a,150] Among these, light has attracted special attention because remotely triggered release of drugs including photosensitizers, chemotherapeutic drugs, genes, and proteins with an external modulator can provide high spatiotemporal control.[qv: 22a,150c] This ability can maximize drug efficacy by improving local drug accumulation efficiently while minimizing the toxic side effects of the drug. However, the technical difficulties to minimize drug release when they are not triggered, to systematically study biocompatibility and biodegradability of nanomaterials and surface‐modified materials, to increase long‐term storage and stability, and to minimize nonspecific binding to tissues exist for clinical applications.[qv: 150a,c] One of the practical problems is that, in the case of light stimuli, treatment is preferentially activated at a specific location chosen by the operator, since significant energy of light must be concentrated on the nanoparticles, which can be disadvantageous if the location of the target is not visible. In addition, quantitative monitoring of drug release has important implications in decision making for an individualized therapy with an appropriate efficacy. Li et al. synthesized core–satellite ICG/DOX@Gel‐CuS nanoparticles, which are composed of gelatin nanoparticles, near‐infrared fluorochrome ICG, chemo‐drug DOX, and CuS nanoparticles.^151^ The fluorescence of ICG was initially quenched by satellite CuS NPs and increased in proportion to the amount of DOX released by enzyme‐activated degradation in real‐time. Additionally, the photoacoustic signal from CuS nanoparticles was utilized for real‐time tracking and degradability of core–satellite nanoparticles in a tumor site. Plasmonic nanoparticles have been used as nanoantennas or contrast agents due to the strong absorption and scattering properties, including dark‐field microscopy, SERS, multiphoton microscopy, which could be combined with drug delivery, PTT and PDT. Therefore, these efforts to harmonize drug delivery, imaging and therapy aspects together while addressing practical issues will accelerate the progress of future drug delivery systems and facilitate practical applications of these nanostructures in the near future.

## 4 Conclusion and Outlook

Numerous types of plasmonic nanomaterials have been developed to enable scientists to explore biomedical applications that require the photothermal effect for diagnosis and treatment. The intelligent design and precise synthesis of plasmonic nanoparticles can greatly alter and improve their absorption property that is directly related to heat generation; therefore, the light‐to‐heat conversion efficiency can be enhanced with tunable resonance wavelength. Furthermore, heat activation can be remotely controlled by an external light source. Upon illumination, the nanoparticles act as nanosized heaters to precisely deliver highly localized thermal energy to the desired area with high spatiotemporal resolution. Moreover, the amount and degree of delivered heat can be controlled by the light intensity. Although many researchers have demonstrated the properties of newly synthesized plasmonic photothermal nanomaterials and validated their applications in biomedical science, the widespread use of these nanomaterials for practical applications is limited by the absence of synthetic methods for targeting nanostructures in high yield with high precision and reproducibility. Further fundamental studies of these nanomaterials are required to optimize the photothermal conversion process, and particle surface modification with surface ligands and surface‐protection molecules is also a key process for the use of these particles for in vivo applications.

The photothermal effect is a phenomenon that photon energy is converted into thermal energy by plasmonic materials, and there are few energy loss pathways other than heat dissipation for nanoparticles upon light absorption.^152^ Efficient and uniform heating of individual nanoparticles is highly important for the homogeneity of the reaction; therefore, obtaining nanoparticles with a narrow absorbance line width, high extinction, and low polydispersity in both size and shape is crucial for maximized and controlled photothermal responses. Similarly, because the pharmacokinetic aspects (absorption, distribution, metabolism, and excretion) are also highly dependent on the structure and composition of nanoparticles,^148^, ^153^ such highly engineered nanoparticles are needed to minimize the dosage to prevent unwanted side effects or toxicity, and to achieve long circulation time, target‐specific accumulation, and efficient clearance from body, especially for imaging, therapy, and drug delivery applications. It is also highly important to study and develop optimized surface conjugation and encapsulation strategies for the different photothermal nanoparticles to create new types of nanostructures with unique properties such as specific and sensitive biorecognition, biocompatibility, solubility, enhanced optical signaling, and particle stability.

The fundamental theories and equations defining light absorption and scattering, heat generation, and heat conduction are well established; however, realization into practical systems and devices is still challenging and unaddressed. Although temperature rise of the nanoparticle surface can provide a unique tool for localized manipulation with high spatiotemporal control of many biological processes at the molecular, cellular, and even tissue level, this property has not been fully utilized in many biomedical applications that require precisely controlled thermal energy (e.g., ultrafast DNA melting,^154^ nanoscale membrane melting,^155^ molecular or drug release,^7^ or nanoscale protein denaturation^156^). Because it is difficult to measure the actual temperature of a nano and microscale materials, whereas macroscale temperature can be easily measured by traditional methods such as thermocouples, infrared cameras fluoroptic thermometers,[qv: 66a] it is essential to develop measurement tools and methods for the characterization of nano and microscale temperature surrounding nanoparticles. Recently, there have been many efforts to develop a thermometer capable of measuring the temperature of nano and microenvironments and materials, so‐called nanothermometry.^157^ Contact (scanning thermal microscopy), noncontact (infrared thermography, interferometric method and Raman spectroscopy) and semicontact (thermometry based on luminescent nanoparticles showing temperature‐dependent luminescence—organic dyes, polymers, green fluorescent proteins, quantum dots, upconversion nanoparticles, and nanodiamonds) methods are used for nanothermometry.^158^ Among them, scanning microscopy‐based techniques showed the best spatial resolution of ≈10 nm, and the interferometry showed the best thermal resolution of ≈0.1 mK.^157^ The contact and noncontact nanothermometry methods can measure local temperature mainly for 2D surface and microstructured samples, with the instrumental limitations including complexity in set‐ups and difficulty in defining refractive index of the fluid (interferometric) and the temperature limitations at high temperatures due to a black‐body radiation background (above ≈1000 K) and low temperatures due to the weak population of vibrational modes (Raman spectroscopy).^157^ To measure temperatures with 3D spatial resolution in biological environments, highly inhomogeneous with respect to composition, refractive index and pH, the luminescent nanoparticle‐based semicontact methods can be a better option with the capability of remote‐sensing obtained by excitation and emission from the nanoparticles traveling through the sample.^157^ The development of nontoxic nanomaterials that can more effectively absorb and emit light in the NIR‐I to NIR‐II region will allow nanothermometry to be utilized in the real in vivo applications. It is also necessary to study and compare light‐to‐heat conversion efficiency, time‐dependent trajectory and mechanism, heat transfer to the medium, and the limit of temperature rise for nanostructures with varying shapes and sizes.^159^

Designing and realizing cost‐effective, reliable, automated, portable, and miniaturized instrumentation is another emerging issue for personalized healthcare and point‐of‐care platforms. As microoptics, miniaturized optomechanical components, light sources, and interconnecting technology with smart devices rapidly mature, plasmonic photothermal nanomaterials will be expanded to and provide new opportunities in diverse biomedical applications.

## Conflict of Interest

The authors declare no conflict of interest.
